# Expected outcomes of stratified post‐donation testing in whole blood donation in England: A discrete event simulation modeling study

**DOI:** 10.1111/trf.18165

**Published:** 2025-02-25

**Authors:** Hongchao Qi, Angela M. Wood, Stephen Kaptoge, Amy McMahon, Susan Mehenny, Nathalie Kingston, Willem H. Ouwehand, John Danesh, David J. Roberts, Emanuele Di Angelantonio, Lois G. Kim

**Affiliations:** ^1^ BHF Cardiovascular Epidemiology Unit, Department of Public Health and Primary Care University of Cambridge Cambridge UK; ^2^ NIHR Blood and Transplant Research Unit in Donor Health and Behaviour Cambridge UK; ^3^ Victor Phillip Dahdaleh Heart and Lung Research Institute University of Cambridge Cambridge UK; ^4^ British Heart Foundation Centre of Research Excellence University of Cambridge Cambridge UK; ^5^ Health Data Research UK Cambridge Wellcome Genome Campus and University of Cambridge Cambridge UK; ^6^ Cambridge Centre of Artificial Intelligence in Medicine Cambridge UK; ^7^ British Heart Foundation Data Science Centre Health Data Research UK London UK; ^8^ NHS Blood and Transplant Wakefield UK; ^9^ NIHR BioResource Cambridge University Hospitals NHS Foundation Trust, Cambridge Biomedical Campus Cambridge UK; ^10^ Department of Haematology, School of Clinical Medicine University of Cambridge, Cambridge Biomedical Campus Cambridge UK; ^11^ Department of Haematology, Heart and Lung Research Institute University of Cambridge, Cambridge Biomedical Campus Cambridge UK; ^12^ NHS Blood and Transplant, Cambridge Biomedical Campus Cambridge UK; ^13^ Department of Haematology Cambridge University Hospitals, Cambridge Biomedical Campus Cambridge UK; ^14^ University College London Hospitals London UK; ^15^ Department of Human Genetics Wellcome Sanger Institute Hinxton UK; ^16^ NHS Blood Transplant, John Radcliffe Hospital Oxford UK; ^17^ Radcliffe Department of Medicine University of Oxford Oxford UK; ^18^ Health Data Science Centre, Human Technopole Milan Italy

**Keywords:** blood donation, discrete event simulation, low hemoglobin deferral, post‐donation testing

## Abstract

**Background:**

In England, blood donors with low hemoglobin concentration are deferred following on‐session testing to prevent donations below regulatory thresholds, thereby protecting donors' health and blood supply quality. However, deferrals are costly, time‐consuming and may discourage donors. Post‐donation testing (PDT), where hemoglobin levels are measured after donation, offer potential alternatives as used in some European countries.

**Study Design and Methods:**

We compared four PDT strategies to the current approach: (A) no on‐session testing, (B) on‐session testing if low hemoglobin at previous visit, (C) on‐session testing if low/medium hemoglobin at previous visit, all with delayed reinvitation if low hemoglobin at previous donation, and (D) on‐session testing if low/medium hemoglobin at previous visit without delayed reinvitations.

We employed discrete event simulation modeling, informed by data collected from 16,941 donors returning under the current strategy in England, to simulate and compare total donations, under‐threshold donations, and deferrals for each strategy over 18 months.

**Results:**

Strategy A eliminated deferrals but led to increased under‐threshold donations compared to the current strategy in men (6.5% vs. 2.3%) and women (11.8% vs. 4.5%).

Strategies B–D reduced deferrals rates for men (1.0%–3.7% vs. 5.5%) and women (2.2%–6.3% vs. 8.9%) but showed slightly higher under‐threshold donations in men (3.0%–5.1% vs. 2.3%) and women (5.3%–8.8% vs. 4.5%). Strategies with more on‐session testing had lower under‐threshold donations.

**Discussion:**

PDT strategies incorporating on‐session testing for low/medium hemoglobin at previous visits could reduce deferrals while maintaining a low proportion of under‐threshold donations, thereby balancing donor safety with operational efficiency.

**Abbreviations**
DES: discrete event simulation
NHSBT: National Health Service Blood and Transplant
PDT: post‐donation testing
SD: standard deviation
STRIDES: strategies to improve donor experiences

## 1 INTRODUCTION

The National Health Service Blood and Transplant (NHSBT) is the sole provider of blood for NHS England, with a mandate for ensuring the health and safety of donors while maintaining a sufficient, resilient, and high‐quality blood supply. To protect donors from anemia, NHSBT currently screens hemoglobin levels during donation sessions, deferring those whose hemoglobin levels fall below the regulatory thresholds (135 g/L for men and 125 g/L for women).^1^, ^2^ Deferrals are intended to serve as preventive measures, as blood donors are particularly vulnerable to iron deficiency due to iron loss associated with donation.^3^ However, the current testing strategy in NHSBT results in a high number of donors deferred due to low hemoglobin,^4^ which can discourage donors and so may reduce donor retention over time.^5^, ^6^, ^7^, ^8^ Furthermore, frequent on‐session deferrals create inefficiencies, consuming valuable resources—such as time, funds, and venue capacity—affecting both NHSBT and donors.^9^

A potential approach to reduce on‐session deferrals due to low hemoglobin is to use post‐donation testing (PDT), where hemoglobin levels are measured after donation with a blood sample taken during the donation session. This approach is already in use in several European countries including Belgium, Denmark, France, and Sweden.^10^ As previously shown,^11^ one potential additional advantage of adopting a PDT strategy is to use a donor's hemoglobin level at the previous donation to calculate a personalized interval, providing some certainty that the donor will be eligible to donate based on hemoglobin recovery after donation. However, implementing such personalized strategy poses practical challenges, requiring individual‐level predictions for hemoglobin levels and customized inter‐donation intervals. Additionally, the approach may have some psychological impact on donors, with those given shorter intervals potentially feeling pressured to reattend donation sessions, while those given longer intervals potentially experiencing concerns about their health.^11^

To address these challenges, a simplified PDT strategy with choice of inter‐donation intervals dependent on strata of hemoglobin levels may offer a more practical solution to test donors safely while improving the efficiency of collection. In this study, we used discrete event simulation (DES) modeling based on hypothetical blood donor population in England to evaluate four stratified PDT strategies. To further safeguard donor health under PDT strategies and reduce instances of under‐threshold donations, we also considered options that combined PDT with on‐session testing for a subset of donors.

## 2 STUDY DESIGN AND METHODS

### 2.1 Study design

To inform the DES modeling, we used data from the Strategies to Improve Donor Experiences (STRIDES) PDT sub‐study, a large observational study embedded into the STRIDES trial (November 2019 to November 2022, Research Ethics Committee reference: 18/EE/0284) and NIHR STRIDES BioResource (Research Ethics Committee reference: 17/EE/0025 and 17/EE/0230).^12^ During the study period (October 2020 to November 2022), donors who consented to participate in the STRIDES PDT sub‐study received NHSBT's routine screening for donation eligibility, that is, measurement of hemoglobin via copper sulphate test, followed by capillary HemoCue test for those who failed the initial test. Additionally, donors recruited in the PDT sub‐study were followed over an 18‐month period, and a blood sample was taken after each donation to measure hemoglobin levels using a Sysmex XN‐9100 hematology analyzer (Sysmex UK Limited, Milton Keynes, UK) at The Doctors Laboratory (TDL), London.

### 2.2 Hemoglobin screening strategies

#### 2.2.1 NHSBT current strategy

**FIGURE 1:**
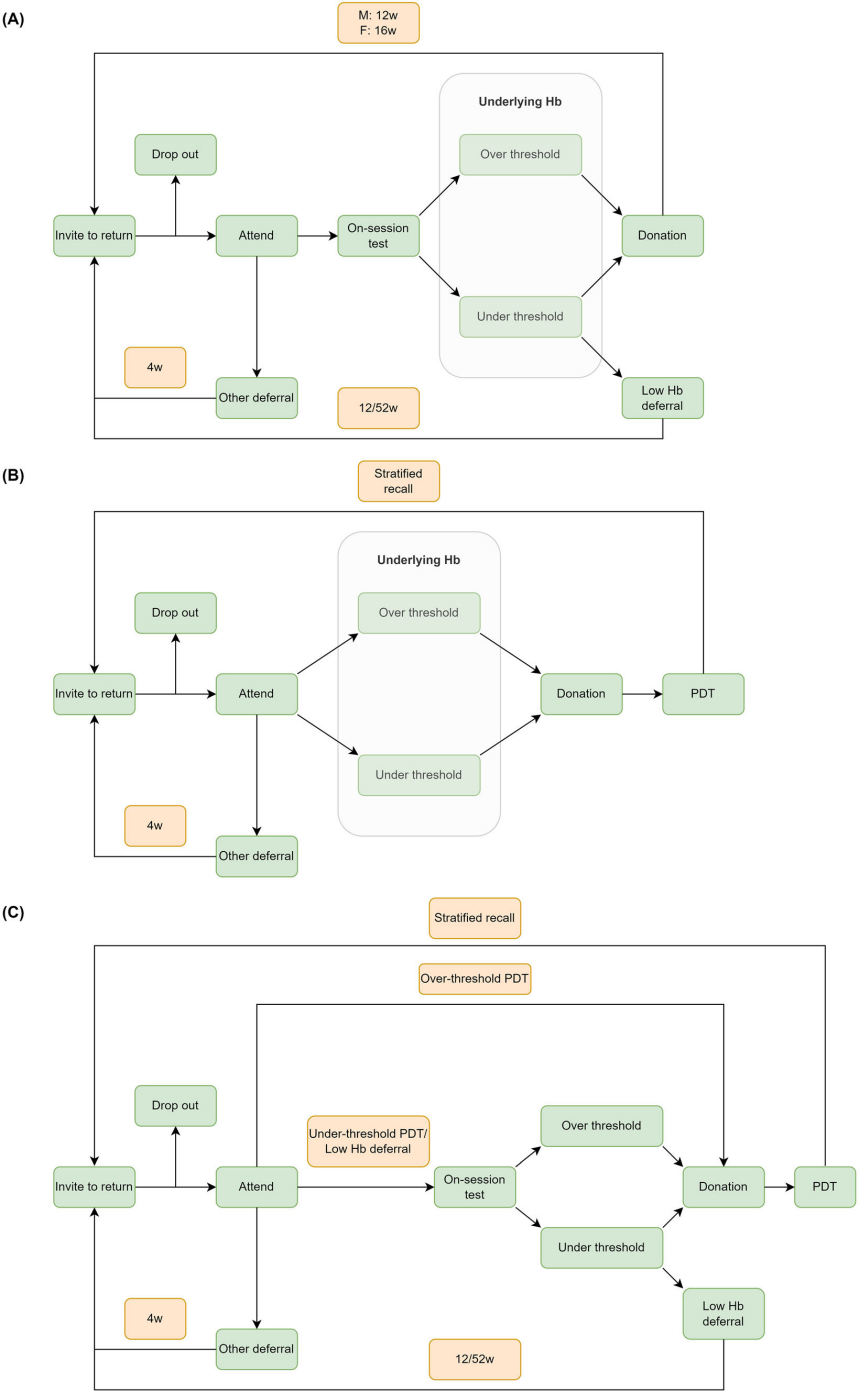
Flowcharts of different strategies, (A) the current strategy, (B) the PDT‐only strategy, and (C) the PDT with limited on‐session testing strategy. [Color figure can be viewed at wileyonlinelibrary.com]

In England, all whole blood donors undergo an on‐session hemoglobin test before each donation. This comprises a copper sulphate test for finger‐prick capillary blood, followed by a spectrophotometric test (HemoCue AB, Ängelholm, Sweden) for finger‐prick capillary blood if the donor fails the copper sulphate test.^13^, ^14^ The regulatory thresholds of hemoglobin to donate are 135 g/L (men) and 125 g/L (women). Donors who pass the hemoglobin test are permitted to donate and are invited to return after 12 weeks (men) or 16 weeks (women). Those failing the on‐session test with borderline hemoglobin—defined as 125–134 g/L in men and 115–124 g/L in women—are deferred for 12 weeks, while donors with very low hemoglobin (<125 g/L in men and <115 g/L in women) are deferred for 52 weeks. Donors deferred for other reasons, including medical reasons, recent travel, and administrative reasons, are invited to return after 4 weeks (Figure 1A).

#### 2.2.2 Post‐donation testing strategies

We evaluated two types of PDT strategies: a PDT‐only strategy without on‐session hemoglobin testing (Figure 1B) and a combined PDT strategy with limited on‐session hemoglobin testing (Figure 1C).

In the PDT‐only strategy, the inter‐donation interval was determined by the post‐donation hemoglobin level categorized into sex‐specific groups as very low, low, medium, and high (Table 1 and Figure 1B), each with a corresponding inter‐donation interval. While this strategy eliminates on‐session low hemoglobin deferrals, it may increase under‐threshold donations. To address this, we considered limited on‐session hemoglobin testing for specific donor groups: (1) those who previously donated below a certain threshold, and (2) those previously deferred for low hemoglobin (Figure 1C).

**TABLE 1:** Percentages of donors categorized by hemoglobin level for men and women.

Sex	Hemoglobin group (g/L)	Percentage (%)
Men	Very low (<125)	0.2 (17/8680)
	Low (125–134)	3.0 (260/8680)
	Medium (135–144)	19.8 (1719/8680)
	High (≥145)	77.0 (6684/8680)
Women	Very low (<115)	0.4 (33/8261)
	Low (115–124)	6.2 (512/8261)
	Medium (125–134)	29.1 (2404/8261)
	High (≥135)	64.3 (5312/8261)

Using the hemoglobin categories from Table 1 and the flowcharts in Figure 1, we constructed four distinct PDT strategies (A to D) combining extended inter‐donation intervals for low‐hemoglobin donors and limited on‐session testing based on previous hemoglobin level (Table 2).

**TABLE 2:** Details of the strategies in this study.

Strategy	Hemoglobin group	Inter‐donation interval (weeks)	On‐session test (yes/no)
Current	Very low	52^a^	✓
	Low	12^a^	✓
	Medium	12 (men); 16 (women)	✓
	High	12 (men); 16 (women)	✓
Strategy A	Very low	52	×
	Low	26	×
	Medium	12 (men); 16 (women)	×
	High	12 (men); 16 (women)	×
Strategy B	Very low	52^b^	✓
	Low	12/26^c^	✓
	Medium	12 (men); 16 (women)	×
	High	12 (men); 16 (women)	×
Strategy C	Very low	52^b^	✓
	Low	12/26^c^	✓
	Medium	12 (men); 16 (women)	✓
	High	12 (men); 16 (women)	×
Strategy D	Very low	52^b^	✓
	Low	12/12^c^ (men); 12/16^c^ (women)	✓
	Medium	12 (men); 16 (women)	✓
	High	12 (men); 16 (women)	×

^a^ No donation: next reinvitation time determined based on hemoglobin at on‐session test before low hemoglobin deferral.

^b^ Donors with very low hemoglobin level (either based on on‐session test or PDT) will be reinvited after 52 weeks.

^c^ Reinvitation interval R1/R2. R1 applies to donors in whom an on‐session test indicates low hemoglobin at current visit, triggering low hemoglobin deferral, reinvitation interval R1 (=12 weeks for all strategies) and on‐session test at next visit. R2 applies to donors who donate under threshold at current visit (i.e., low hemoglobin status identified by post‐donation testing), triggering reinvitation interval R2 (varies by strategy) and on‐session test at next visit.

### 2.3 Statistical analysis

#### 2.3.1 Discrete event simulation modeling

To simulate and evaluate the proposed strategies, we adapted a previously published DES model.^11^ The model simulated donation outcomes and associated costs for each PDT strategy, incorporating estimates from the STRIDES PDT sub‐study on: (1) attendance‐related events such as dropout, low hemoglobin deferrals, and other deferrals; (2) time from invitation to attendance; and (3) hemoglobin levels at return visits.

To estimate probabilities of the attendance‐related events, we calculated the observed proportions of such events in the PDT sub‐study (Table S1). Since nearly all donors with known hemoglobin levels above the threshold proceeded to donate, the model assumed all donors with over‐threshold hemoglobin levels will donate. For donations with missing hemoglobin data, we assumed the same over‐ to under‐threshold donation ratio as observed to estimate the probability of low hemoglobin deferral when hemoglobin levels fall below the threshold.

Using flexible parametric survival models with four knots, we estimated the time from invitation to attendance separately for men and women, adjusting for baseline covariates^15^ (Table S2). The models were adopted from previous DES models,^11^ with additional knots added to accommodate a longer time period in the current analyses.

Hemoglobin levels at return visits were modeled using sex‐specific linear mixed models with predictors including time since the index donation, baseline hemoglobin, baseline donor age, blood type, ethnicity (white vs. non‐white) and a donor‐specific random intercept (Table S3).

To validate the DES model, we compared numbers of predicted events over 18 months from modeling the current strategy to actual observed events.

#### 2.3.2 Simulation settings and outputs

We implemented DES modeling by drawing 10,000 donors, sampled with replacement, from the baseline data of the STRIDES PDT sub‐study. Each of the four PDT strategies was evaluated separately for men and women over an 18‐month period, tracking key donation events, including the number of donations over and under the regulatory thresholds, low hemoglobin deferrals, other deferrals, and on‐session tests. Cost per donation for each strategy were calculated based on the unit costs described in the previous study.^11^ We derived 95% uncertainty intervals (UIs) of the above outputs from 500 probabilistic sensitivity analysis samples drawn from the joint distribution of all parameters in the model.^16^

#### 2.3.3 Strategies evaluation

To evaluate each strategy's performance, we constructed a utility function that weights total donations against two adverse events: under‐threshold donations and low hemoglobin deferrals. Details on the specification of preference weights (i.e., trade‐off parameters) for under‐threshold donations ($T_{\mathrm{UD}}$) and low hemoglobin deferrals $\left(T_{\mathrm{LD}}\right)$ are presented in the Supporting Information. Briefly, $T_{\mathrm{UD}}$ and $T_{\mathrm{LD}}$ represent numbers of donations the blood service is willing to lose to avoid one under‐threshold donation and one low hemoglobin deferral, respectively. For example, if both preference weights are 20, the blood service is willing to lose 20 donations to avoid one under‐threshold donation and 20 donations to avoid one low hemoglobin deferral. A higher utility indicates a better‐performing strategy, for a given particular set of preferences between total number of donations, under‐threshold donations and low hemoglobin deferrals. We did not report apparent utilities for different strategies in isolation as they have limited practical implications. Instead, the probability that a given PDT strategy is better than the current strategy was derived from the simulation results (i.e., the proportion of simulations where the utility of the PDT strategy is higher than that of the current strategy) for performance evaluation.

There are about 1.4 million whole blood donations in England every year.^17^ Based on the STRIDES PDT data, the male to female ratio of blood donations equates to 1.50 (i.e., 23,140 male:15,406 female donations). Therefore, we assumed that there were around 0.84 million whole blood donations from men and around 0.56 million whole blood donations from women in England in 2022. We used the preceding numbers and the simulation results to quantify the potential impact of nationwide implementation of PDT strategies in England.

Finally, we conducted a sensitivity analysis investigating the impact of employing HemoCue testing for on‐session testing in both universal testing and PDT strategies B to D. Attendance‐related parameters relating to HemoCue testing were derived from the COMPARE study,^13^ which included HemoCue measurements for all participants. Details can be found in the Supporting Information (Supplement Section 7).

Statistical analyses were performed using R version 4.4.2 (R Core Team, 2022).

## 3 RESULTS

### 3.1 Donors' characteristics

In total, 16,941 donors (8680 men, 8261 women) were recruited in the PDT sub‐study from October 2020 to November 2022. Mean (SD) ages of men and women were 48.7 (14.2) and 46.1 (14.0) years respectively, with majority of participants being white ethnicity (90.5% for men, 92.8% for women; Table 3). Almost all the donors (98.9% men, 98.5% women) donated at the baseline visit, of whom 2.0% of male donors and 4.5% of female donors donated under the sex‐specific regulatory thresholds for hemoglobin level. In the 2 years before the baseline visit, 5918 male donors (68.2%) and 5086 female donors (61.6%) had successfully donated.

**TABLE 3:** Baseline characteristics of blood donors in the STRIDES PDT sub‐study from October 2020 to November 2022.

Characteristics	Men (*n* = 8680)	Women (*n* = 8261)
Age, mean (SD)	48.7 (14.2)	46.1 (14.0)
Ethnicity, *N* (%)		
White	7957 (91.7)	7666 (92.8)
Other	723 (8.3)	595 (7.2)
Blood group, *N* (%)		
A+	2293 (26.5)	2239 (27.1)
A−	660 (7.7)	835 (10.1)
B+	647 (7.5)	573 (6.9)
B−	231 (2.7)	252 (3.0)
O+	3037 (34.9)	2783 (33.7)
O−	1421 (16.3)	1535 (18.6)
AB+	319 (3.7)	8 (0.1)
AB−	72 (0.8)	36 (0.4)
Attendance‐related events, *N* (%)		
Donation	8587 (98.9)	8137 (98.5)
Low hemoglobin deferral	67 (0.8)	100 (1.2)
Failed donation	23 (0.3)	22 (0.3)
Other deferral	2 (0)	2 (0)
Other	1 (0)	0 (0)
Hemoglobin (g/L), mean (SD)	153 (10)	139 (9)
Under‐threshold donations, *N* (%)	177 (2.0)	372 (4.5)
Donations 1–10 g/L under the threshold, *N* (%)	172 (2.0)	361 (4.4)
Donations >10 g/L under the threshold, *N* (%)	5 (<0.1)	11 (0.1)
Donated in the last 2 years, *N* (%)	5918 (68.2)	5086 (61.6)

During the 18‐month follow‐up period, there were 28,456 and 20,001 return visits among men and women, respectively (median inter‐donation interval: 15 weeks for men and 19 weeks for women; Table S4). Among the return visits, 23,140 (81.3%) and 15,406 (77.0%) resulted in donation for men and women respectively. Overall, 39.6% of the donations for men and 38.4% of the donations for women had missing information on hemoglobin level. The baseline characteristics were balanced for donors with and without missing hemoglobin measures for both men and women implying random missingness (Table S5).

### 3.2 Discrete event simulation

Our DES model showed good internal validity, providing confidence in its ability to accurately simulate current blood donation practices (Table S6). Results for the PDT strategies are shown in Table 4. Across all PDT strategies, the proportion of over‐threshold donations increased in all PDT strategies relative to the current strategy, with percentages ranging from 93.0% to 93.8% for men and 88.2% to 89.0% for women, compared to 92.2% and 86.6% under the current strategy. Low hemoglobin deferral rates also decreased under all PDT strategies, from 0% to 3.7% for men and 0% to 6.3% for women, versus 5.5% and 8.9% in the current approach.

**TABLE 4:** Numbers and percentages (95% uncertainty intervals) of attendance‐related events per 1000 donors and costs per donation (95% uncertainty intervals) followed over an 18‐month period of different strategies based on the STRIDES PDT sub‐study.

Sex	Strategy	Number of events				Percentage of events				Cost per donation (GBP)
		Over‐threshold donations	Under‐threshold donations	Low hemoglobin deferrals	On‐session tests^a^	Over‐threshold donations	Under‐threshold donations	Low hemoglobin deferrals	On‐session tests^a^	
Men	Current^b^	2601	65	155	2821	92.2	2.3	5.5	100	27.03
	Strategy A^c^	2527 (2435, 2610)	177 (141, 209)	‐	0^d^	93.5 (92.3, 94.9)	6.5 (5.1, 7.7)	‐	0^d^	26.20^e^
	Strategy B	2557 (2475, 2647)	140 (119, 171)	28 (16, 42)	115 (102, 127)	93.8 (92.6, 94.9)	5.1 (4.4, 6.1)	1.0 (0.6, 1.6)	4.2 (3.8, 4.7)	26.31 (26.26, 26.36)
	Strategy C	2574 (2463, 2677)	83 (66, 102)	92 (68, 114)	703 (674, 741)	93.6 (92.6, 94.8)	3.0 (2.4, 3.7)	3.3 (2.4, 4.1)	25.6 (24.9, 26.2)	26.59 (26.50, 26.66)
	Strategy D	2609 (2520, 2712)	91 (67, 114)	105 (79, 134)	726 (689, 752)	93.0 (91.2, 94.3)	3.3 (2.4, 4.0)	3.7 (2.8, 4.8)	25.9 (25.2, 26.7)	26.62 (26.53, 26.73)
Women	Current^b^	1773	92	182	2047	86.6	4.5	8.9	100	27.39
	Strategy A^c^	1700 (1601, 1789)	226 (192, 264)	‐	0^d^	88.2 (86.0, 90.3)	11.8 (9.7, 14.0)	‐	0^d^	26.20^e^
	Strategy B	1731 (1663, 1805)	172 (148, 196)	43 (25, 58)	168 (151, 184)	89.0 (87.5, 90.6)	8.8 (7.7, 10.0)	2.2 (1.3, 3.0)	8.6 (7.8, 9.4)	26.43 (26.34, 26.50)
	Strategy C	1748 (1668, 1827)	104 (81, 126)	119 (94, 143)	780 (745, 813)	88.7 (87.1, 90.4)	5.3 (4.1, 6.4)	6.0 (4.8, 7.3)	39.6 (38.8, 40.4)	26.89 (26.76, 27.03)
	Strategy D	1773 (1707, 1858)	111 (89, 138)	127 (96, 149)	800 (760, 837)	88.2 (86.6, 90.3)	5.5 (4.5, 6.8)	6.3 (4.8, 7.4)	39.8 (38.9, 40.5)	26.93 (26.77, 27.04)

^a^ All new donors would undergo on‐session hemoglobin tests.

^b^ Observed results from the STRIDES PDT sub‐study.

^c^ There are no low hemoglobin deferrals in a PDT‐only strategy.

^d^ There are no on‐session tests in a PDT‐only strategy.

^e^ The cost per donation is fixed in strategy A since there are no on‐session tests or low hemoglobin deferrals.

In terms of safety, under‐threshold donations were slightly higher across all PDT strategies compared to the current strategy, affecting both men (3.0%–6.5% vs. 2.3%) and women (5.3%–11.8% vs. 4.5%). Strategy A showed the highest percentages of under‐threshold donations (6.5% for men and 11.8% for women), while Strategy C had the lowest (3.0% for men and 5.3% for women).

Moreover, the number and percentage of on‐session tests required were significantly lower in all PDT strategies for existing donors than in the current approach, highlighting a potential efficiency gain with PDT. All new donors would undergo on‐session hemoglobin tests under any testing strategy. The mean cost per donation was similar across different PDT strategies and was generally lower than in the current strategy. Among the PDT strategies, Strategy A had the lowest mean cost per donation since there are no on‐session tests or low hemoglobin deferrals, while under‐threshold donations do not carry an explicit financial cost to the blood service provider.

### 3.3 Evaluation of overall strategy performance

**FIGURE 2:**
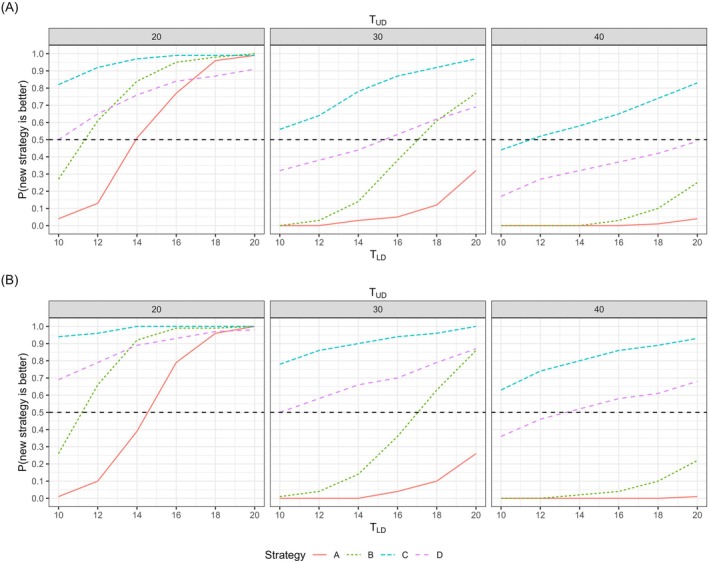
Probabilities that each PDT strategy is better than the current strategy in terms of utility for (A) men and (B) women, where *T*_*UD*_ represents the relative weighting between under‐threshold donations and total donations, and *T*_*LD*_ represents the relative weighting between low hemoglobin deferrals and total donations. [Color figure can be viewed at wileyonlinelibrary.com]

The probabilities of each PDT strategy (A to D) outperforming the current strategy in terms of utility are presented in Figure 2. When both preference weights are set to 20, all PDT strategies (A to D) have a greater than 90% probability of achieving higher utility than the current strategy for both men and women. However, with a higher preference weight for under‐threshold donations (i.e., 40) and a lower weight for low hemoglobin deferrals (i.e., 10), all PDT strategies have less than a 50% probability of surpassing the current strategy, indicating that the current approach is preferred. The only exception is Strategy C in women, which has a 63% probability of outperforming the current strategy.

Among the PDT strategies, Strategy C performed the best in most preference combinations because it has the lowest proportion of under‐threshold donations, and the preference weight of under‐threshold donations is always larger than or equal to that of low hemoglobin deferrals. Strategy A performed the worst for most preference combinations since it has the highest proportion of under‐threshold donations. Strategy B is only better than Strategy D when the preference weight of under‐threshold donations is low and that of low hemoglobin deferrals is high due to its high proportion of under‐threshold donations and low proportion of low hemoglobin deferrals.

Projected annual event numbers in England by strategy for men and women are presented in Table 5. If Strategy C is implemented, we could expect 9000 fewer over‐threshold donations alongside 6000 more under‐threshold donations, and 20,000 fewer low hemoglobin deferrals per annum in male donors. In female donors, we could expect 7000 fewer over‐threshold donations alongside 4000 more under‐threshold donations and 20,000 fewer low hemoglobin deferrals per annum.

**TABLE 5:** Projected annual numbers of events in England by strategy for men and women (1.4 million donations).

Sex	Strategy	Number of attendance‐related events (thousands)			Total visits^a^
		Over‐threshold donations	Under‐threshold donations	Low hemoglobin deferrals	
Men	Current	820	20	49	889
	Strategy A	797	55	‐	852
	Strategy B	807	44	9	859
	Strategy C	811	26	29	866
	Strategy D	823	28	33	884
Women	Current	532	28	55	615
	Strategy A	511	68	‐	579
	Strategy B	521	52	13	585
	Strategy C	525	32	35	592
	Strategy D	533	33	38	604

^a^ Non‐hemoglobin‐related deferrals are not considered in the calculation.

Results for PDT strategies with HemoCue‐only on‐session tests are provided in Table S8. PDT with on‐session HemoCue testing performed better than universal HemoCue testing for all preference combinations for both men and women due to large numbers of low hemoglobin deferrals in the universal HemoCue testing strategy. However, the PDT strategies with on‐session HemoCue testing are worse than their counterparts (Strategies B to D).

## 4 DISCUSSION

In this study, we propose and evaluate several PDT strategies using simulation modeling based on a large study of returning whole blood donors. Our findings suggest that while the PDT‐only strategy enhances efficiency in blood donation practice it may compromise donor safety, by substantially increasing the number of under‐threshold donations. In contrast, a PDT strategy incorporating limited on‐session testing for donors with low/medium hemoglobin levels effectively reduces under‐threshold donations while maintaining reasonable efficiency. Moreover, if there are under‐threshold donations, then a full blood count is available to the donor clinical team to manage the donor by allocating an appropriate interval until the next donation or referral to the donor's medical practitioner if needed. In contrast, under the current system of on‐session testing, donors giving an under‐threshold donation cannot be identified or actively managed. This suggests that adopting a PDT strategy with limited on‐session testing may be a good alternative to the current practice in England.

The most important advantage of PDT strategies is their potential to greatly reduce or even eliminate on‐session testing and low hemoglobin deferrals for blood donors. However, the preferred strategy for the blood service depends on a pre‐determined trade‐off between total donations, under‐threshold donations and low hemoglobin deferrals, which could be selected based on expert opinions via, for example, the Delphi method. Beyond these trade‐off considerations, reducing low hemoglobin deferrals could yield additional benefits for the blood service and donors in terms of time, money, and venue capacity.^11^, ^18^ In the long term, it may facilitate maintaining donors' return rate, not only by reducing low hemoglobin deferrals but also by reducing the need for finger‐prick tests and/or venipuncture when attending the donation session.^7^ Taken together, these changes may increase the resilience of the blood supply chain.

Our simulation model reflects the current on‐session testing strategy used in NHSBT and the STRIDES trial, which uses a copper sulphate test followed by a capillary HemoCue test for those who fail the copper sulphate test. However, the COMPARE study indicated that this approach may lead to increased under‐threshold donations compared to a single universal capillary HemoCue test.^13^ Our modeling suggests that implementing a PDT strategy where donors offered HemoCue‐only on‐session testing may reduce under‐threshold donations relative to the current strategy, although it may also increase substantially low hemoglobin deferrals.

A notable strength of this study is that the STRIDES PDT sub‐study is, to our knowledge, the only observational study designed to investigate the feasibility of PDT strategies in the English national blood service. The availability of repeated post‐donation hemoglobin measures allowed us to develop models that informed the simulations. We cannot make direct comparisons of hemoglobin measures between post‐donation Sysmex (the gold standard) and on‐session HemoCue based on the current data, but we used the same model to model hemoglobin recovery in the simulations. Therefore, the misclassification due to on‐session HemoCue was accounted for in the modeling. Moreover, this large observational study provides high quality data to inform pragmatic estimates of probabilities relating to attendance outcomes, including donations and deferrals, as well as time intervals from invitation to attendance. We conducted an internal validation exercise for the DES model, which demonstrated broad similarity between numbers of simulated attendance‐related events and numbers of observed attendance‐related events assuming random missingness. Finally, we conducted a comprehensive comparison of different strategies accounting for a range of outcomes, including attendance‐related events, cost per donation, and utility combining efficiency and safety. Note that not all the cost information related to low hemoglobin deferrals (e.g., wasted venue capacity and donors' time) was included in the cost calculation. A pilot study on the PDT strategy that will collect more comprehensive cost information is currently being planned.

However, this study has some potential limitations. First, hemoglobin measures were missing for more than 30% of donations in the data because some research blood samples were not taken after donation due to unforeseen practice changes during the COVID‐19 pandemic. We assumed these were missing at random, and that the linear mixed models fitted provided valid inference for the parameters of interest.^19^ Second, we did not report under‐threshold donations at borderline low hemoglobin levels (e.g., 134 g/L for men, 124 g/L for women) and those at more extreme values separately. In reality, a donation just below the threshold is less likely to harm the donor's health.^14^ Third, we only evaluated a limited set of possible strategies which may have excluded the optimal number of strata, cutoffs to define strata (either to define reinvitation times or who is offered on‐session testing) and exact reinvitation times. Finally, we have not evaluated the generalizability of our results for settings outside NHSBT.

In conclusion, this study provides evidence supporting the future implementation of PDT strategies in NHSBT and blood services in other countries. A well‐designed PDT with limited on‐session testing for donors at highest risk of under‐threshold donation has the potential to improve service efficiency while maintaining the safety of the current practice of blood donation in England.

## FUNDING INFORMATION

This work/Angela M. Wood was supported by the NIHR Research Professorship (NIHR303137). John Danesh was supported by a British Heart Foundation Personal Chair. Emanuele Di Angelantonio holds an NIHR Senior Investigator Award. Participants in the STRIDES trial were recruited with the active collaboration of NHS Blood and Transplant England (www.nhsbt.nhs.uk), which has supported field work and other elements of the trial. The academic coordinating center at the Department of Public Health and Primary Care at the University of Cambridge received core support from the NIHR Blood and Transplant Research Unit (NIHR203337), British Heart Foundation (RG/13/13/30194; RG/18/13/33946) and NIHR Cambridge Biomedical Research Centre (BRC‐1215‐20014). This work was supported by core funding from the NIHR Blood and Transplant Research Unit (NIHR203337), British Heart Foundation (RG/F/23/110103), NIHR Cambridge Biomedical Research Centre (NIHR203312), BHF Chair Award (CH/12/2/29428), Cambridge BHF Centre of Research Excellence (RE/24/130011, RE/18/1/34212) and by Health Data Research UK, which is funded by the UK Medical Research Council, Engineering and Physical Sciences Research Council, Economic and Social Research Council, Department of Health and Social Care (England), Chief Scientist Office of the Scottish Government Health and Social Care Directorates, Health and Social Care Research and Development Division (Welsh Government), Public Health Agency (Northern Ireland), British Heart Foundation and the Wellcome Trust (HDRUK2023.0028). The views expressed are those of the author(s) and not necessarily those of the NIHR, NHSBT or the Department of Health and Social Care.

## CONFLICT OF INTEREST STATEMENT

John Danesh serves on scientific advisory boards for AstraZeneca, Novartis, and UK Biobank, and has received multiple grants from academic, charitable and industry sources outside of the submitted work.

## Supporting information


**Data S1:** Supporting Information.
