# Supplementary material for: Expected outcomes of stratified post‐donation testing in whole blood donation in England: A discrete event simulation modeling study
Source: Transfusion. 2025 Feb 25;65(4):696–707. doi: 10.1111/trf.18165 (PMC12005575; doi:10.1111/trf.18165)
Supplement: Supplementary file 1 — Data S1: Supporting Information. [file TRF-65-696-s001.docx]

**Supplement for ‘Expected outcomes of stratified post-donation testing in whole blood donation in England: a discrete event simulation modelling study’**

1. **Structure of the STRIDES trial**


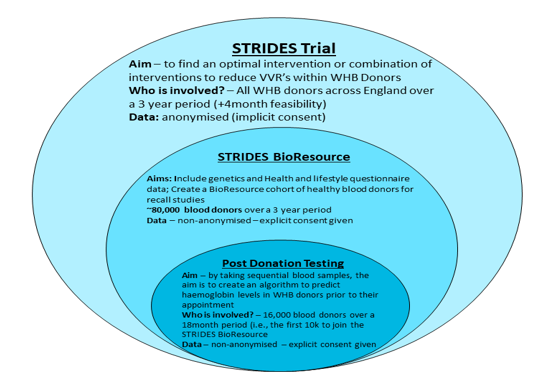


Figure S1. Relationship between the STRIDES trial and NIHR STRIDES BioResource and PDT sub-studies

1. **DES model parameterization**

Table S1. Transition parameters of attendance-related events in the 18-month period

| Transition | Men (N=8680) | | Women (N=8261) | |
| --- | --- | --- | --- | --- |
|  | Observed transitions | P (transition) | Observed transitions | P (transition) |
| Drop out | 630/8680 | 0.0726 | 915/8261 | 0.1108 |
| Other deferrals | 3972/28456 | 0.1396 | 3088/20001 | 0.1544 |
| Low haemoglobin deferrals given below-threshold haemoglobin | 1344/1909 | 0.7040 | 1507/2265 | 0.6653 |

Table S2. Parameter estimates of 4-knot parametric survival models for men and women

| Parameter | Estimate (95% confidence intervals) | |
| --- | --- | --- |
|  | Men | Women |
| Age (year) | 1.005 (1.004, 1.006) | 1.005 (1.004, 1.006) |
| Ethnicity |  |  |
| White | Ref. | Ref. |
| Other | 1.029 (0.954, 1.111) | 1.003 (0.893, 1.127) |
| Blood group |  |  |
| A+ | Ref. | Ref. |
| A- | 0.994 (0.931, 1.061) | 0.987 (0.920, 1.058) |
| B+ | 1.044 (0.975, 1.117) | 1.058 (0.974, 1.148) |
| B- | 0.997 (0.901, 1.104) | 1.093 (0.977, 1.223) |
| O+ | 1.056 (1.014, 1.101) | 1.027 (0.978, 1.079) |
| O- | 1.043 (0.993, 1.095) | 1.057 (1.000, 1.117) |
| AB+ | 1.072 (0.983, 1.168) | 1.409 (0.749, 2.650) |
| AB- | 1.041 (0.877, 1.235) | 1.005 (0.761, 1.326) |
| Previous low haemoglobin deferrals |  |  |
| 0 | Ref. | Ref. |
| 1 | 1.062 (0.976, 1.156) | 1.037 (0.959, 1.122) |
| ≥2 | 0.983 (0.731, 1.323) | 1.088 (0.840, 1.410) |
| Previous donations |  |  |
| 0-2 | Ref. | Ref. |
| 3-4 | 1.281 (1.236, 1.327) | 1.368 (1.301, 1.438) |
| ≥5 | 1.808 (1.609, 2.032) | 4.558 (1.140, 18.230) |
| Baseline haemoglobin | 1.004 (0.987, 1.022) | 0.988 (0.966, 1.010) |
| Hazard function parameters |  |  |
| Gamma0 | -5.683 (-6.016, -5.350) | -5.021 (-5.380, -4.661) |
| Gamma1 | 0.604 (0.551, 0.657) | 0.480 (0.431, 0.529) |
| Gamma2 | -0.054 (-0.073, -0.034) | -0.178 (-0.201, -0.155) |
| Gamma4 | 0.005 (-0.049, 0.058) | 0.270 (0.222, 0.319) |
| Gamma5 | 0.128 (0.063, 0.192) | -0.043 (-0.092, 0.005) |
| Gamma6 | -0.086 (-0.119, -0.053) | -0.084 (-0.112, -0.056) |

Table S3. Parameter estimates of linear mixed models for men and women

| Parameter | Estimate (95% confidence intervals) | |
| --- | --- | --- |
|  | Men | Women |
| Intercept | 3.4304 (3.1461, 3.7146) | 3.1972 (2.8971, 3.4973) |
| Age (year) | -0.0027 (-0.0039, -0.0014) | 0.0048 (0.0034, 0.0061) |
| Ethnicity |  |  |
| White | Ref. | Ref. |
| Other | -0.0933 (-0.1766, -0.01) | -0.0986 (-0.2133, 0.0162) |
| Blood group |  |  |
| A+ | Ref. | Ref. |
| A- | -0.0475 (-0.1171, 0.0221) | 0.0384 (-0.0315, 0.1083) |
| B+ | 0.007 (-0.0654, 0.0794) | 0.0677 (-0.0128, 0.1483) |
| B- | 0.1082 (-0.0011, 0.2175) | -0.0744 (-0.1849, 0.0362) |
| O+ | 0.0437 (-0.004, 0.0877) | 0.0757 (0.0269, 0.1246) |
| O- | 0.0592 (0.0067, 0.1117) | 0.0797 (0.0242, 0.1352) |
| AB+ | -0.0027 (-0.0953, 0.090) | -0.1998 (-0.8746, 0.475) |
| AB- | -0.0949 (-0.2885, 0.0987) | 0.2723 (-0.0012, 0.5458) |
| Time since index visit (week) | 0.0008 (0.002, 0.0013) | 0.0022 (0.0015, 0.0029) |
| Baseline haemoglobin | 0.7615 (0.7435, 0.7795) | 0.7201 (0.6986, 0.7417) |
| Variances |  |  |
| Between-subject variance | 0.2856 | 0.2821 |
| Error variance | 0.4317 | 0.4200 |

1. **Return visits in the follow-up**

Table S4. Details of return visits in the 18-month follow-up period for men and women

|  | Men | Women |
| --- | --- | --- |
| Number of return visits | 28456 | 20001 |
| Length of follow-up (years), median (IQR) | 1.2 (0.9, 1.4) | 1.2 (0.9, 1.3) |
| Inter-donation interval (weeks), median (IQR) | 15 (12.9, 19.7) | 19 (16.9, 26.0) |
| Number of return visits per donor, N (%) |  |  |
| 0 | 630 (7.3) | 915 (11.1) |
| 1 | 1042 (12.0) | 1377 (16.7) |
| 2 | 1315 (15.1) | 1794 (21.7) |
| 3 | 1535 (17.7) | 2128 (25.8) |
| 4 | 1803 (20.8) | 1679 (20.3) |
| ≥5 | 2355 (27.1) | 368 (4.5) |
| Visits with failed donation or other deferral, N (%) | 3972 (14.0) | 3088 (15.4) |
| Of remaining visits: |  |  |
| Donation, N (%) | 23140 (81.3) | 15406 (77.0) |
| Low haemoglobin deferral, N (%) | 1344 (4.7) | 1507 (7.5) |
| Of donations: |  |  |
| Known haemoglobin, N (%) | 13977 (60.4) | 9492 (61.6) |
| Missing haemoglobin, N (%) | 9163 (39.6) | 5914 (38.4) |
| Haemoglobin (g/L), mean (SD) | 151 (9) | 138 (9) |
| Under-threshold donations, N (%) | 341 (2.4) | 467 (4.9) |
| Donations 1-10 g/L under the threshold, N (%) | 332 (2.4) | 452 (4.8) |
| Donations >10 g/L under the threshold, N (%) | 9 (<0.1) | 15 (0.1) |
| Of low haemoglobin deferrals: |  |  |
| Known haemoglobin, N (%) | 1306 (97.2) | 1474 (97.8) |
| Missing haemoglobin, N (%) | 38 (2.8) | 33 (2.2) |
| Haemoglobin (g/L), mean (SD) | 128 (7) | 118 (6) |

Table S5. Distributions of baseline characteristics for donors without and with missing haemoglobin measures in the follow-up by sex

| Sex | Characteristics | No missing | Missing | P value |
| --- | --- | --- | --- | --- |
| Men | N | 2917 | 5763 |  |
|  | Age, mean (SD) | 48.5 (14.6) | 48.8 (13.9) | 0.36 |
|  | Ethnicity, N (%) |  |  | 0.71 |
|  | White | 2679 (91.8) | 5278 (91.6) |  |
|  | Other | 238 (8.2) | 485 (8.4) |  |
|  | Blood group, N (%) |  |  | 0.07 |
|  | A+ | 821 (28.1) | 1472 (25.5) |  |
|  | A- | 215 (7.4) | 445 (7.7) |  |
|  | B+ | 208 (7.1) | 439 (7.6) |  |
|  | B- | 79 (2.7) | 152 (2.6) |  |
|  | O+ | 1025 (35.1) | 2012 (34.9) |  |
|  | O- | 429 (14.7) | 992 (17.2) |  |
|  | AB+ | 117 (4.0) | 202 (3.5) |  |
|  | AB- | 23 (0.8) | 49 (0.9) |  |
| Women | N | 3572 | 4689 |  |
|  | Age, mean (SD) | 45.8 (13.9) | 46.3 (14.0) | 0.11 |
|  | Ethnicity, N (%) |  |  | 0.50 |
|  | White | 3323 (93.0) | 4343 (92.6) |  |
|  | Other | 249 (7.0) | 346 (7.4) |  |
|  | Blood group, N (%) |  |  | 0.08 |
|  | A+ | 1023 (28.6) | 1216 (25.9) |  |
|  | A- | 356 (10.0) | 479 (10.2) |  |
|  | B+ | 267 (7.5) | 306 (6.5) |  |
|  | B- | 103 (2.9) | 149 (3.2) |  |
|  | O+ | 1154 (32.3) | 1629 (34.7) |  |
|  | O- | 649 (18.2) | 886 (18.9) |  |
|  | AB+ | 2 (0.1) | 6 (0.1) |  |
|  | AB- | 18 (0.5) | 18 (0.4) |  |

1. **Internal validity of the DES model**

Table S6. Internal validity of the DES model by numbers of attendance-related events per 1000 donors by sex

|  | Attendance-related events | | | |  |
| --- | --- | --- | --- | --- | --- |
|  | Over-threshold donations | Under-threshold donations | Low haemoglobin deferrals | Other deferrals | Total |
| Men |  |  |  |  |  |
| Observed | 2601 | 65 | 155 | 458 | 3278 |
| Predicted | 2612 | 60 | 146 | 455 | 3273 |
| Difference (%) | +0.4 | -7.7 | -5.8 | -0.7 | -0.2 |
| Women |  |  |  |  |  |
| Observed | 1773 | 92 | 182 | 374 | 2421 |
| Predicted | 1766 | 86 | 166 | 358 | 2376 |
| Difference (%) | -0.4 | -6.6 | -8.8 | -4.3 | -1.7 |

1. **Specification of preference weights for under-threshold donations and low haemoglobin deferrals**

To evaluate the strategies in the simulation, we constructed a utility function by combining total number of donations, number of under-threshold donations and number of low haemoglobin deferrals. The utility function is given by

$U=N_{TD}-T_{UD}\times N_{UD}-T_{LD}\times N_{LD}$,

where $U$ is the utility, $N_{TD}$ is the total number of donations, $T_{UD}$ is the preference weight of under-threshold donation, $N_{UD}$ is the number of under-threshold donations, $T_{LD}$ is the preference weight of low haemoglobin deferral, and $N_{LD}$ is the number of low haemoglobin deferrals. $T_{UD}$ and $T_{LD}$ represent numbers of donations that the blood service is willing to lose to avoid one under-threshold donation and one low haemoglobin deferral, respectively.

In this study, $T_{UD}$ and $T_{LD}$ were specified based on the observed ratios of the total number of donations to the number of under-threshold donations and the number of low haemoglobin deferrals. In the STRIDES PDT sub-study, the ratios were (2601+65)/65= 41.0 and (2601+65)/155=17.2 respectively for men, and (1773+92)/92= 20.3 and (1773+92)/182= 10.2 respectively for women. In the simulation, we constructed the utility functions based on the combinations of $T_{UD}$ ranging from 20 to 40 at an increment of 10 and $T_{LD}$ ranging from 10 to 20 at an increment of 2. For example, if $T_{UD}=40$ and $T_{LD}=10$, the blood service is willing to lose 40 donations to avoid one under-threshold donation and 10 donations to avoid one low haemoglobin deferral.

1. **Effect of delayed recall on attendance-related events**

In the main text, we show that although the PDT-only strategy showed its advantage in efficiency, it performed poorly in terms of safety, resulting in a substantial increase of the percentage of under-threshold donations. The percentage of under-threshold donations remained high even when the inter-donation interval was lengthened to 52 weeks for blood donors with low haemoglobin levels. Simulation results are presented in **Table S7**. In contrast, providing on-session haemoglobin tests to donors with low/medium haemoglobin levels can effectively reduce under-threshold donations according to the main results.

Table S7. Numbers and percentages of attendance-related events for strategy A and strategy A with delayed recall interval for low haemoglobin group by sex

| Sex | Strategy | Number of events | | Percentage of events | |
| --- | --- | --- | --- | --- | --- |
|  |  | Over-threshold donations | Under-threshold donations | Over-threshold donations | Under-threshold donations |
| Men | Strategy A | 2527 (2435, 2610) | 177 (141, 209) | 93.5 (92.3, 94.9) | 6.5 (5.1, 7.7) |
|  | PDT-only strategy with delayed recall | 2460 (2356, 2560) | 147 (121, 177) | 94.4 (93.2, 95.3) | 5.6 (4.7, 6.8) |
| Women | Strategy A | 1700 (1601, 1789) | 226 (192, 264) | 88.2 (86.0, 90.3) | 11.8 (9.7, 14.0) |
|  | PDT-only strategy with delayed recall | 1625 (1549, 1704) | 185 (154, 213) | 89.8 (88.1, 91.5) | 10.2 (8.5, 11.9) |

^a^ There are no low haemoglobin deferrals in PDT-only strategies.

1. **Simulation results for strategies with HemoCue on-session testing**

We investigated the impact of HemoCue-only on-session testing and PDT strategies with HemoCue on-session testing on the attendance-related outcomes via simulation based on transition parameters derived from the COMPARE data. The only change to the PDT strategies in the main text was changing the on-session testing to universal capillary HemoCue test, we did not run the simulation for PDT-only strategy because no on-session testing is involved. We named the counterparts of the PDT strategies in the main text as ‘Strategy B2’, ‘Strategy C2’, and ‘Strategy D2’. Simulation results, i.e., point estimates and 95% uncertainty intervals (UIs) from PSA, are presented in **Table S8**.

Table S8. Numbers and percentages (95% UIs) of attendance-related events per 1000 donors and costs per donation (95% UIs) followed over an 18-month period of different strategies with HemoCue on-session testing

|  |  | Number of events | | | | Percentage of events | | | | |  |
| --- | --- | --- | --- | --- | --- | --- | --- | --- | --- | --- | --- |
| Sex | Strategy | Over-threshold donations | Under-threshold donations | Low haemoglobin deferrals | On-session tests | Over-threshold donations | Under-threshold donations | Low haemoglobin deferrals |  | On-session tests | Cost per donation (GBP) |
| Men | HemoCue-only | 2270 (2168, 2364) | 45 (32, 58) | 475 (435, 522) | 2790 (2688, 2869) | 81.4 (79.7, 82.9) | 1.6 (1.1, 2.1) | 17.0 (15.6, 18.6) |  | 100 | 28.38 (28.20, 28.59) |
|  | Strategy B2 | 2544 (2416, 2665) | 137 (114, 161) | 40 (25, 57) | 126 (111, 142) | 93.5 (92.2, 94.5) | 5.0 (4.2, 6.0) | 1.5 (0.9, 2.1) |  | 4.6 (4.1, 5.2) | 26.35 (26.29, 26.41) |
|  | Strategy C2 | 2503 (2401, 2576) | 76 (59, 97) | 173 (143, 205) | 766 (732, 802) | 91.0 (89.6, 92.2) | 2.8 (2.2, 3.6) | 6.3 (5.2, 7.4) |  | 27.8 (27.1, 28.8) | 26.88 (26.77, 27.01) |
|  | Strategy D2 | 2540 (2445, 2627) | 81 (63, 97) | 186 (154, 223) | 788 (753, 834) | 90.5 (88.7, 91.8) | 2.9 (2.3, 3.5) | 6.6 (5.5, 8.0) |  | 28.1 (27.3, 29.2) | 26.92 (26.80, 27.07) |
| Women | HemoCue-only | 1467 (1373, 1535) | 45 (32, 57) | 538 (488, 595) | 2050 (1959, 2144) | 71.6 (69.6, 73.4) | 2.2 (1.5, 2.8) | 26.2 (24.5, 28.3) |  | 100 | 29.77 (29.48, 30.13) |
|  | Strategy B2 | 1717 (1646, 1806) | 162 (139, 187) | 72 (51, 93) | 196 (176, 216) | 88.0 (86.0, 89.7) | 8.3 (7.1, 9.7) | 3.7 (2.6, 4.8) |  | 10.0 (9.1, 11.1) | 26.57 (26.46, 26.69) |
|  | Strategy C2 | 1662 (1569, 1747) | 80 (64, 96) | 258 (222, 298) | 880 (833, 930) | 83.1 (81.3, 85.3) | 4.0 (3.2, 4.8) | 12.9 (11.2, 14.5) |  | 44.0 (42.9, 45.0) | 27.67 (27.46, 27.87) |
|  | Strategy D2 | 1679 (1604, 1738) | 81 (65, 98) | 272 (235, 320) | 900 (844, 956) | 82.7 (80.2, 84.9) | 4.0 (3.2, 4.8) | 13.4 (11.7, 15.3) |  | 44.3 (43.3, 45.6) | 27.73 (27.52, 27.97) |

Utilities summarising performance for each strategy were also calculated in this session. The probability that a given PDT strategy is better than the HemoCue-only strategy (i.e., the proportion of simulations where the utility of the PDT strategy is higher than that of the current strategy) was derived from the simulation results, see **Figure S2**. All the PDT strategies are better than the HemoCue-only strategy for all the combinations of $T_{UD}$ and $T_{LD}$ due to large numbers of low haemoglobin deferrals in the HemoCue-only strategy. However, the above PDT strategies are worse than their counterparts in the main text (strategies B-D) because of high low haemoglobin deferrals.


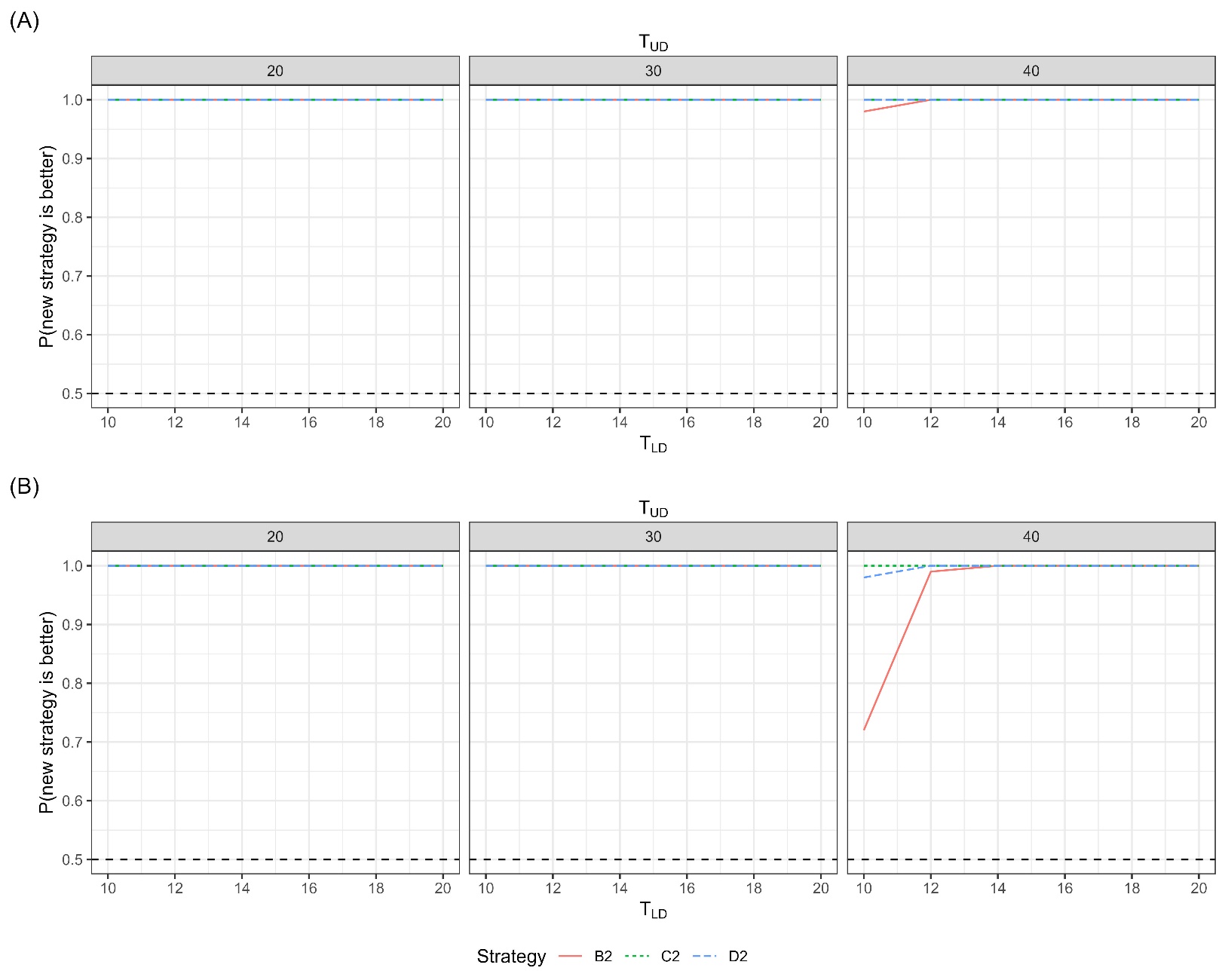


Figure S2. Probabilities that each PDT strategy is better than the HemoCue-only strategy in terms of utility for (A) men and (B) women, where $T_{UD}$ represents the relative weighting between under-threshold donations and total donations, and $T_{LD}$ represents the relative weighting between low haemoglobin deferrals and total donations.
